# Upper airways changes associated with orthodontic molar distalization by Pendulum appliance in adolescent patients: a multicenter retrospective cephalometric study

**DOI:** 10.1186/s13005-024-00461-x

**Published:** 2024-10-16

**Authors:** Marco Serafin, Gero Kinzinger, Jan Hourfar, Valentina Mantellini, Rosamaria Fastuca, Alberto Caprioglio

**Affiliations:** 1 Department of Biomedical Sciences for Health, University of Milan, Via Mangiagalli 31, 20133 Milan, Italy; 2 Department of Orthodontics, Saarland University, Homburg, Germany; 3 Department of Biomedical, Surgical and Dental Sciences, University of Milan, Milan, Italy; 4 IRCSS Cà Granda, Ospedale Maggiore Policlinico, Milan, Italy; 5 Private Practice, Lugano, Swiss Switzerland

**Keywords:** Class II, Molar distalization, Pendulum appliance, Upper airways

## Abstract

**Objectives:**

The purpose of this multicenter retrospective study was to perform a two-dimensional analysis of upper airway changes in adolescent patients following molar distalization with the Pendulum appliance.

**Materials and methods:**

The study involved the cephalometric analysis of 88 patients, retrospectively categorized into two groups: skeletal Class II with a dental Class II molar relationship (36 patients, mean age 12.6 ± 1.1 years) and skeletal Class I with a dental Class II molar relationship (54 patients, mean age 12.3 ± 1.2 years). Changes were observed using lateral radiographs before (T0) and after Pendulum appliance removal (T1); treatment time averaged 7 months. Upper airways were subsequently analyzed by tracing lateral radiographs. The Shapiro-Wilk test showed a normal distribution of the data, therefore parametric tests were used for statistical analysis. Intragroup changes between T0 and T1 were evaluated using paired t-tests, and intergroup differences were assessed using independent student t-tests; statistical significance was set at 0.05.

**Results:**

Statistically significant differences were observed in the skeletal measurements that characterized both groups, particularly in ANB and Wits appraisal, at T0 (*P* < 0.001). After molar distalization, Class I and Class II groups reported no statistically significant differences with changes almost equal to zero between timepoints (*P* > 0.05). Additionally, intergroup comparisons of airway changes at T1 did not show statistically significant differences (*P* > 0.05).

**Conclusions:**

The Pendulum appliance does not significantly change the upper airway dimensions in Class I and Class II malocclusion patients, thereby minimizing potential respiratory risks.

## Introduction

Upper molar distalization as the initial step in a non-extraction treatment protocol, aims to either regain or create space in the posterior region of the dental arch [1, 2]. In skeletal Class I patients with a Class II molar relationship, space must be regained following mesial migration of the molars, which may occur for example due to early tooth loss. In skeletal class II patients with a prognathic maxilla and dental Class II molar relationship the creation of additional space through molar distalization facilitates the dental camouflage of Class II malocclusion. This sagittal lengthening of the dental arch in the posterior direction promotes reactive growth in the maxillary tuberosity region [3, 4].

Molar distalization represents the initial phase of non-extraction treatment focused on establishing Class I molar and canine relationships, particularly through the use of non-compliance devices [5]. While the majority of intraoral and extraoral devices are effective for achieving molar distalization, their success is often highly dependent on patient compliance [6]. Among the various non-compliance distalization devices, the Pendulum appliance is one of the most widely used and extensively studied [7].

The standard Pendulum appliance was first described by Hilgers in 1992 [8]. Different modifications, such as the Pendulum K by Kinzinger have been documented in the literature [9–14].

In recent years, the orthodontic treatment of patients with obstructive sleep apnoea syndrome (OSAS) has gained increasing interest. Pediatric OSAS involves upper airway obstruction during sleep, characterized by snoring, increased respiratory effort, increased upper airway resistance and collapse of the pharyngeal space [15]. Children with this pathological condition remain largely underdiagnosed, affecting neurocognitive development and resulting in behavioral problems [16]. Class II malocclusion is a dysmorphic condition associated with an increased risk of breathing disorders [17]. The use of mandibular advancement devices, as well as interceptive orthodontics approaches, has been shown to be extremely effective in improving outcomes in patients with OSAS [18].

A 2016 Cochrane systematic review and meta-analysis on oral devices and functional orthopedic therapy concluded that there was insufficient evidence to support or refute the effectiveness of these therapies in the treatment of OSAS in children [19]. Recently, a 2023 meta-analysis reported positive effects on the severity of pediatric OSAS with treatment through functional orthodontics [20].

While the effects of mandibular advancement on the upper airway are well-documented, the same cannot be said for molar distalization; only a few studies have reported the effect of molar distalization on the upper airway, but the results remain unclear. As a result, there is a gap in the literature investigating effects of molar distalization, particularly in growing patients with dental Class II malocclusion, where an extraction protocol- and thus, a constriction of maxillary dental arch, is avoided.

Therefore, the aim of the present study was to investigate whether the Pendulum appliance causes changes in the upper airway following upper molar distalization, and if the therapeutic effects differ between Class I and Class II patients due to variations in maxillary prognathism.

## Materials and methods

### Sample selection

The protocol of this retrospective study was reviewed and approved by the Ethical Committee of the University of Milan, Italy (approval number 87/22); all the procedures followed the World Medical Organization Declaration of Helsinki. All patients gave written permission and signed informed consent, permitting the use of their anonymized data.

The sample size was calculated based on a preliminary pilot sample of 5 patients, with the primary outcome being the amount of molar distalization (mean difference of 4.0 mm). To achieve a power of β = 0.80 with α = 0.05, a minimum of at least 32 subjects per group was necessary.

To analyze changes in the upper airways, cephalometric tracings of 88 growing patients (43 males and 45 females) were retrospectively analyzed. This included 36 patients with skeletal Class II malocclusion and 54 patients with skeletal Class I, the latter representing the control group. The mean age of the patients was 12.6 ± 1.1 years and 12.3 ± 1.2 years for Class II and Class I groups, respectively. The mean duration of distalization therapy was 238 days.

Patients were selected according to the following inclusion criteria:

- Skeletal Class I or Class II malocclusion with a bilateral full-class or end-to-end Class II molar relationship;
- Peak growth spurt, classified as stage C3 according to the cervical vertebrae maturation method [21];
- Distalization treatment using the Pendulum appliance;
- High-quality radiographs;
- Treatment duration of less than 12 months, with no other orthodontic or orthopedic treatments during the distalization period.

The analysis of the patients was carried out by evaluating lateral radiographs taken before starting the therapy (T0) and after the removal of the Pendulum device (T1). From the initial sample of 100 consecutively treated patients, 12 were excluded due to insufficient radiographic quality, which prevented accurate analysis and landmark identification, potentially introducing selection and measurements biases.

### Clinical management

All patients were treated with either the modified Hilgers’ Pendulum [8] or the Pendulum K [9–14] devices, which are tooth-borne appliances featuring a Nance button on the palate for intraoral anchorage and titanium-molybdenum springs that provide continuous, and mild forces to achieve upper first molar distorotation and distalization. The appliance was maintained in place until a bilateral super Class I molar relationship was established. The average treatment duration was 7 months. Following the removal of the Pendulum device, a passive Nance button was placed to ensure the maintenance of the achieved distalization, and fixed orthodontic treatment was continued. All cases analyzed in this retrospective study were consecutively treated by two board-certified orthodontists in their respective clinics between 2012 and 2023. No changes to the design of the appliance were made during this period.

### Cephalometric analysis

The cephalometric analysis of the lateral radiographs was conducted using a specialized software (DeltaDent; Outside Format, Pandino, Italy), specifically designe for evaluating thicknesses and distances relating to the upper airways. The following linear measurements were analyzed:

- Ptv-AD: The shortest distance between the pharyngeal adenoid tissue (AD) and the Pterygoid Vertical line (PtV), measured 5 mm above the Posterior Nasal Spine (PNS) [22].
- Upper Nasopharyngeal Space (UNS): The distance between the PNS and the nearest adenoid tissue, measured along a line perpendicular to the Sella-Basion (S-Ba) line passing through the PNS [23].
- Lower Nasopharyngeal Space (LNS): The distance between the PNS and the closest adenoid tissue, measured along the PNS-Ba line [23].
- Upper Adenoid Thickness (UAT): The thickness of the adenoid tissue, measured along the line from the PNS to the midpoint of the line joining the sella (S) with basion (Ba) [24].
- Lower Adenoid Thickness (LAT): The thickness of the adenoid tissue, measured at the level of the PNS-Ba line [24].
- Inferior Pharyngeal Space (IPS): The distance between the posterior pharyngeal wall and the surface of the tongue, measured along a line parallel to the Frankfurt horizontal (FH) plane at the lower level of the second Cervical vertebra (C2) [25].
- Epiglottic Pharyngeal Space (EPS): The distance between the posterior pharyngeal wall and the surface of the tongue, measured along a line parallel to the FH plane at the level of the epiglottic tip [25].

**Fig. 1:**
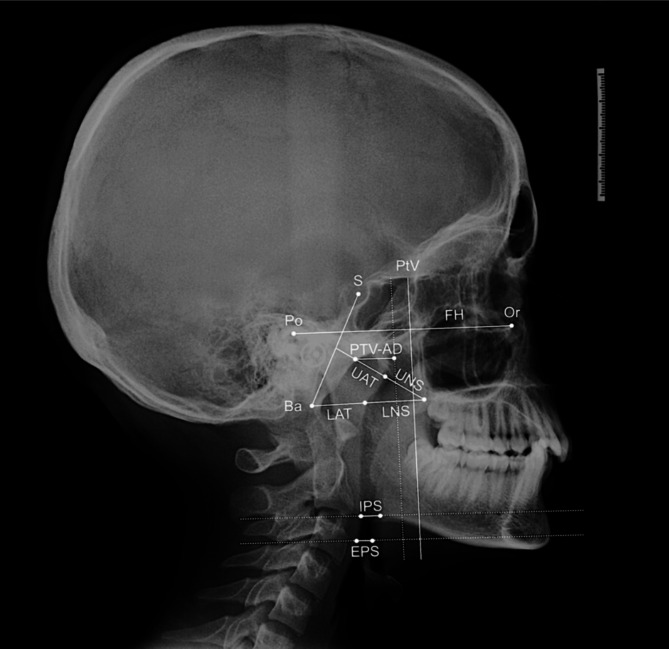
Cephalometric analysis of the airways. Landmarks: Ba, Basion; Po, Porion; S, Sella; Or, Orbitale. Planes: PtV, Pterygoid Vertical plane; FH, Frankfurt plane. Measurements: Ptv-AD, Pterygoid Vertical-Adenoid tissue; UNS, Upper Nasopharyngeal Space; LNS, Lower Nasopharyngeal Space; UAT, Upper Adenoid Thickness; LAT, Lower Adenoid Thickness; IPS, Inferior Pharyngeal Space; EPS, Epiglottic Pharyngeal Space

Figure 1 provides a graphical summary of all the airway measurements analyzed in this study.

### Statistical analysis

SPSS software (version 22.0; IBM, Armonk, NY, USA) was used for the statistical analysis. The Shapiro-Wilk test showed a normal distribution of the data, allowing the use of parametric tests. Means and standard deviations (SD) were calculated for both groups. The Student T-test was used to compare baseline characteristics between the 2 groups. The Paired t-test was used to compare changes within each group between T0 and T1. Additionally, the Student T-test was used to compare differences between groups. Statistical significance was set at 0.05.

The method of moments (MME) variance estimator was used to quantify the method error. Measurements from 10 randomly selected subjects were repeated twice at a one-month interval. The repeated measurements were compared with Student T-test and showed no significant differences.

## Results

### Intergroup comparison at T0

Table 1 presents the means and standard deviations (SDs) of the skeletal and airway measurements for the Class I and Class II groups at T0.

Statistically significant differences were observed in the skeletal measurements that characterize the skeletal pattern of both groups, specifically in ANB and Wits appraisal (*P* < 0.001). No other values were statistically significant when compared between the groups, including growth patterns represented by the Bjork-Jarabak polygon. Differences in this measurement could introduce bias in comparisons due to differential growth.

In the evaluation of airway distances and spaces, it was noted that the distances to the adenoid tissue, specifically UNS and LNS, were on average similar between the Class I and Class II groups at the start of treatment, with no statistically significant differences (*p* > 0.05). Similarly, the thicknesses of the adenoid tissues, represented by UAT and LAT values, did not show significant differences at T0, despite a slight increase in the Class I group (*p* > 0.05). The pharyngeal spaces measured as IPS and EPS, also showed similar values at T0, with no statistical significance between the groups (*p* > 0.05).

**Table 1:** Statistical intergroup analysis of skeletal and airway cephalometric measurements at T0

Skeletal measurements	Class I		Class II		*P*
	Mean	SD	Mean	SD	
SNA (°)	78.89	10.70	81.56	15.02	0.086
SNB (°)	77.50	3.49	77.05	11.08	0.275
ANB (°)	2.72	1.25	4.47	1.82	< 0.001*
Wits (mm)	0.26	0.93	3.45	1.79	< 0.001*
SN^MP (°)	34.05	4.53	33.84	6.57	0.423
Bjork-Jarabak polygon (°)	393.64	4.73	393.72	5.72	0.469
**Airway measurements**					
PtV-AD (mm)	10.23	3.41	11.15	3.64	0.225
UAT (mm)	22.33	3.16	21.78	2.30	0.372
LAT (mm)	23.21	4.24	21.83	3.86	0.121
UNS (mm)	18.13	3.03	18.92	3.37	0.249
LNS (mm)	22.03	4.02	22.85	3.99	0.344
IPS (mm)	10.43	2.61	10.08	2.36	0.519
EPS (mm)	9.79	2.69	9.41	2.20	0.483

* *p* < 0.05

### Intragroup comparison between T0 and T1

Table 2 highlights the differences in the airway values obtained between T0 and T1 for the two groups included in the present study. None of the analyzed measurements showed statistically significant differences, regardless of the group (*p* > 0.05). Both the Class I and Class II groups reported changes that were negligible between the two timepoints.

**Table 2:** Statistical intragroup analysis of airway cephalometric changes between T0 and T1

Airway measurements	Class I					Class II				
	T0		T1		*P*	T0		T1		*P*
	Mean	SD	Mean	SD		Mean	SD	Mean	SD	
PtV-AD (mm)	10.23	3.41	10.85	3.46	0.350	11.15	3.64	11.96	3.79	0.358
UAT (mm)	22.33	3.16	22.16	3.36	0.326	21.78	2.30	21.55	2.27	0.173
LAT (mm)	23.21	4.24	22.95	4.05	0.285	21.83	3.86	21.90	3.43	0.443
UNS (mm)	18.13	3.03	18.43	3.01	0.248	18.92	3.37	19.14	3.56	0.172
LNS (mm)	22.03	4.02	22.22	3.77	0.373	22.85	3.99	22.78	4.01	0.413
IPS (mm)	10.43	2.61	9.99	2.41	0.145	10.08	2.36	9.99	2.33	0.386
EPS (mm)	9.79	2.69	9.41	2.49	0.182	9.41	2.20	9.46	2.31	0.444

### Intergroup comparison between T0 and T1

The comparison of airway changes between the groups following molar distalization, showed no statistical significance (*p* > 0.05). The largest difference between the groups was observed in the EPS values (0.43 mm), whereas the smallest difference was in the UAT values (0.04 mm). None of these changes were considered clinically relevant. The results are presented in Table 3.

**Table 3:** Statistical intergroup analysis of airway cephalometric changes at T1

Airway measurements	Class I		Class II		*P*
	T0-T1		T0-T1		
	Mean	SD	Mean	SD	
PtV-AD (mm)	0.61	2.76	0.82	1.31	0.346
UAT (mm)	-0.18	2.68	-0.22	1.38	0.462
LAT (mm)	-0.25	3.07	0.07	2.71	0.312
UNS (mm)	0.30	3.01	0.22	1.37	0.445
LNS (mm)	0.19	4.02	-0.07	1.98	0.361
IPS (mm)	-0.44	2.83	-0.09	1.91	0.268
EPS (mm)	-0.38	2.85	0.05	2.01	0.226

## Discussion

The impact of orthodontic appliances on airway dimensions is a subject of increasing interest, particularly given concerns about obstructive sleep apnea and other respiratory conditions. The present study aimed to investigate the correlation between adolescent skeletal Class II patients with a dental Class II molar relationship and skeletal Class I patients with a dental Class II molar relationship, all treated with the Pendulum appliance, and the potential changes in the dimensions of the nasopharynx, velopharynx, and oropharynx. The results of the present study did not show any significant changes in airway dimensions within or between the two treatment groups.

A comparison of our findings with existing literature is difficult, since most of the previous studies focused on upper airway analysis following molar class correction through functional treatments instead of molar distalization.

The upper airway, especially the oropharynx and nasopharynx, plays a crucial role in breathing. Thus, any substantial anatomical changes in the maxilla, as well as changes in the positions of teeth and adjacent soft tissues, could theoretically influence the dimensions and functionality of the upper airway.

A recent study reported the effects of molar distalization using skeletal anchorage in growing Class II patients, where 3D CBCT analysis showed an increase in the upper airway volume after treatment; however, the increase was not statistically significant compared with a control group [26]. On the contrary, a two-dimensional analysis using lateral cephalograms reported a slight but no statistically significant decrease in upper airway dimensions following molar distalization with infrazygomatic crest skeletal anchorage for Class II molar correction [27]. Unfortunately, this study included subjects aged 15 to 30 years, making direct comparison with the growing patients in the present study difficult. Another study carried out a three-dimensional analysis of airway space after molar distalization in adult patients using skeletal anchorage observed a non-significant reduction in oropharyngeal airway space [28].

The analysis of previous results is varied and often inconclusive. Some research findings suggest negligible or minor increases in airway dimensions, while others report a potential reduction that could potentially influence respiratory function. These discrepancies can be attributed to various factors, including differences in study design, sample size, measurement techniques, and patient variability. As a result, there is currently no consensus within the orthodontic community regarding the extent and clinical significance of upper airway changes following molar distalization in adolescent Class I and Class II patients.

Another factor that should be considered is the vertical dimension and its control during distalization therapy. Uncontrolled skeletal bite opening can result in potential alteration of the upper airway and tongue posture. Although the Pendulum appliance does not appear to increase the vertical dimension significantly, it should be used with caution in patients with a long-face pattern, or preferably replaced with more biomechanically advantageous devices [29, 30].

Lastly, a possible explanation for the lack of changes in airway dimensions may be related to the initial diagnosis of patients undergoing molar distalization. The upper molars move backward into the space offered by the maxillary tuberosity or occupy the space left by the extraction of upper second or third molars. As a result, it is plausible that the molars move within a bony boundary, with no posterior displacement of soft tissues. Additionally, it is conceivable that changes in molar position could influence tongue posture.

In this context, it is clinically relevant that changes in the functional space (both volume and morphology) can influence tongue posture. Since Class II malocclusions are sometimes associated with tongue dysfunction, the re-education of the latter could potentially alter the pharyngeal space and, consequently, reduce the dimensions for the upper airway [31]. Establishing a physiological swallowing function, including a harmonious tongue resting position, is more likely to be achieved with molar distalization than with premolar extraction, due to arch length development. This is important for long term stability. In contrast, during extraction therapy where functional space is reduced, a lower tongue resting position may occur. Therefore, further studies on extraction patients are necessary to investigate the effects of molar distalization on the posterior airway space.

## Limitations

Addressing orthodontic treatments in growing patients poses challenges, particularly in distinguishing whether observed changes are a result of the treatment or to the patient’s intrinsic growth. This is arguably the most significant limitation and source of bias in both the present and previous studies.

Furthermore, this study relied on two-dimensional lateral cephalometric analysis, whereas the most accurate and widely accepted method for cephalometry is three-dimensional CBCT. Although 3D methods offer greater accuracy and effectiveness, they are subject to methodological variables correlated to the extended image acquisition process, such as changes in the patient’s head position and jaw movements such as swallowing during the exam.

## Conclusions

Based on the results, molar distalization using the Pendulum appliance does not appear to significantly alter upper airway dimensions in Class I or Class II malocclusion patients. This is reassuring for both clinicians and patients, as the risk of unintended respiratory consequences appears minimal. Nonetheless, continuous monitoring and a personalized approach to treatment planning are always recommended. Future studies should aim to further validate these findings, perhaps looking at long-term effects or the cumulative impact of combined orthodontic treatments on airway dimensions and the subsequent development of respiratory disorders.

## Data Availability

No datasets were generated or analysed during the current study.
